# The Role of Movement Kinematics in Facial Emotion Expression Production and Recognition

**DOI:** 10.1037/emo0000835

**Published:** 2021-03-04

**Authors:** Sophie Sowden, Bianca A. Schuster, Connor T. Keating, Dagmar S. Fraser, Jennifer L. Cook

**Affiliations:** 1 Centre for Human Brain Health, School of Psychology, University of Birmingham

**Keywords:** facial emotion expression, emotion recognition, kinematics, speed, face tracking software

## Abstract

The kinematics of peoples’ body movements provide useful cues about emotional states: for example, angry movements are typically fast and sad movements slow. Unlike the body movement literature, studies of facial expressions have focused on spatial, rather than kinematic, cues. This series of experiments demonstrates that speed comprises an important facial emotion expression cue. In Experiments 1a–1c we developed (*N* = 47) and validated (*N* = 27) an emotion-induction procedure, and recorded (*N* = 42) posed and spontaneous facial expressions of happy, angry, and sad emotional states. Our novel analysis pipeline quantified the speed of changes in distance between key facial landmarks. We observed that happy expressions were fastest, sad were slowest, and angry expressions were intermediate. In Experiment 2 (*N* = 67) we replicated our results for posed expressions and introduced a novel paradigm to index communicative emotional expressions. Across Experiments 1 and 2, we demonstrate differences between posed, spontaneous, and communicative expression contexts. Whereas mouth and eyebrow movements reliably distinguished emotions for posed and communicative expressions, only eyebrow movements were reliable for spontaneous expressions. In Experiments 3 and 4 we manipulated facial expression speed and demonstrated a quantifiable change in emotion recognition accuracy. That is, in a discovery (*N* = 29) and replication sample (*N* = 41), we showed that speeding up facial expressions promotes anger and happiness judgments, and slowing down expressions encourages sad judgments. This influence of kinematics on emotion recognition is dissociable from the influence of spatial cues. These studies demonstrate that the kinematics of facial movements provide added value, and an independent contribution to emotion recognition.

Facial emotion expression and recognition play an important role in successful social interaction (Ekman & Friesen, 1975), providing cues about others’ affective states to guide behavior. The last decade has seen increasing application of facial emotion tracking software in research, clinical and educational settings. For example, tracking software is used to dynamically adapt and personalize online learning platforms for education in response to users’ spontaneous expressions (Saneiro et al., 2014; Yang et al., 2018). Facial emotion tracking also holds promise for early diagnosis of various clinical conditions. For instance, autism spectrum disorder (Brewer et al., 2016), Parkinson’s disease (Bologna et al., 2016), and various genetic syndromes (Taggart et al., 2016) have been linked with differences in facial expression. Ongoing efforts to expedite diagnosis using body motion analysis (e.g., Anzulewicz et al., 2016; Zhan et al., 2018) may be improved by including data regarding the extent to which facial expressions are atypical.

It is well established that spatial features of facial expressions play a key role in emotion recognition. A body of work has previously identified key facial action units (actions of groups of muscles) that reliably map on to discrete emotional expressions (Ekman & Friesen, 1977; Frank et al., 1993), while others have shown the relative importance of upper and lower facial areas for the recognition of different emotions (Bassili, 1979; Wegrzyn et al., 2017). For example, mouth-extension actions reliably indicate expressions of happiness, while eyebrow-centered and lip tightening movements indicate expressions of anger and eyebrow-centered and lip corner depressor actions indicate sadness. Moreover, spatial exaggeration of these facial actions leads to increased emotion intensity ratings (Calder et al., 2000; Calder et al., 1997; Hill et al., 2005; Pollick et al., 2003). Thus, if one were to video an actor’s facial expressions of emotion and look at static snapshots, the spatial cues in each snapshot would be good indicators of the corresponding emotional state.

In addition to static, facial expressions also contain dynamic cues (see Dobs et al., 2018 for a review of dynamic face stimuli) including both temporal and kinematic information. Temporal information concerns the temporal order of facial action unit activation. In other words, if one were to video a facial expression, the temporal information comprises the order of the frames within the video. Kinematic information concerns all properties of movement except force and, in the context of the body and face movement literature, typically refers to the speed, acceleration, and jerk (change in acceleration) of movements of parts of the body/face (Cook et al., 2013). Since speed, acceleration, and jerk tend to be highly correlated, kinematic studies often focus on speed as a proxy for kinematic changes in general. Thus, in an expression video, the kinematic cue of interest might be the speed at which an actor moves their mouth from neutral into a full smile.

A small body of work demonstrates that temporal cues play an important role in emotion recognition. For example, across a large number of trials Jack and colleagues (Delis et al., 2016; Jack et al., 2014; Jack & Schyns, 2015) present faces that transition in and out of different patterns of facial action unit activation, and participants are asked to judge the most likely emotion expressed by the face. Jack and colleagues then reverse correlate participants’ categorical emotion responses with the facial action unit activated in the corresponding face. Their analysis demonstrates that not only are specific action units diagnostic of individual emotions (e.g., movement of the eyebrow region aids in distinguishing expressions of anger from disgust) but the temporal order of action unit activation is also crucial for successful emotion identification. Switching the order of key action units within a sequence diagnostic of a specific emotion, significantly impairs categorization of that emotion. Such work demonstrates the importance of the temporal order of activation of facial action units across emotional expressions but does not elucidate whether emotional state impacts on the kinematics (i.e., the temporal activation) of individual facial movements. In other words, do the internal features of the face move faster when an individual feels happy compared to when they feel sad?

To date, studies have not explicitly quantified the contribution of kinematic information to facial emotion expression; for example, to the best of our knowledge, studies have not compared the speed of movement of face features (e.g., mouth opening) between emotions. However, some insight can be gained from the body movement literature. With respect to body movements, evidence suggests that kinematics differ as a function of emotional state: faster body movements are associated with anger and happiness, while slower movements are indicative of sadness (Barliya et al., 2013; Crane & Gross, 2007; Edey et al., 2017; Michalak et al., 2009; Pollick et al., 2001; Roether et al., 2009; Sawada et al., 2003; Schuster et al., 2019). Further insight comes from a small number of studies of facial expressions in which the speed of video or expression-morphing playback has been manipulated and corresponding effects on emotion recognition measured (Fayolle & Droit-Volet, 2014; Kamachi et al., 2013; Pollick et al., 2003). Results from these studies are mixed regarding whether kinematics play a role in emotion perception. Nevertheless, in such paradigms the method of stimulus generation renders movement artificially generated thus these studies cannot inform us about naturally occurring emotion-related kinematic cues. For example, expression morphing is conducted by morphing between static neutral and static facial emotion expressions to create the illusion of a dynamic, moving face. Consequently, the primary aims of the current study were to test whether the speed of facial expression differs as a function of emotion and establish whether this kinematic cue is influential in emotion recognition judgments.

In any study of facial expression, differences that may occur as a function of the context in which the facial expression is produced, should not be overlooked. For instance, the extant literature highlights potential differences between the kinematics of spontaneous, posed and communicative (communicative [for another individual’s benefit] vs. noncommunicative [devoid of social context]) facial expressions. Such differences may be important in real-world applications of facial emotion processing. For example, although emotion-tracking software typically aims to detect spontaneous expressions, much of our knowledge comes from the posed expressions of professional actors, meaning that tracking software may be suboptimally trained with respect to spontaneous expressions. Early evidence suggests posed expressions to be more asymmetrical than spontaneous expressions (Frank et al., 1993). Similarly, direct comparison of posed and spontaneous expressions, using the Facial Action Coding System—a gold-standard taxonomy of facial emotion expressions—has revealed considerable morphological differences (Namba et al., 2017). Thus, at least in terms of spatial presentation, posed and spontaneous expressions are not one and the same. Only a handful of studies have investigated differences in dynamic cues between posed and spontaneous expressions. Evidence suggests that posed and spontaneous expressions can be distinguished based on the timing of expression onset and offset, with posed onset phases being shorter—and thus expressions occurring before those of spontaneous expressions—for both brow (Valstar et al., 2006) and mouth actions (Cohn & Schmidt, 2004; Schmidt et al., 2006). However, to the best of our knowledge, studies have not quantified whether full facial expression kinematics differ between spontaneous and posed emotional expressions. Similarly, although evidence suggests differences in recognition accuracy between communicative and noncommunicative expressions (Brewer et al., 2016), it is unclear whether these relate to differences in the kinematics of the expressions because studies have not mapped expression kinematics in communicative and noncommunicative contexts. Consequently, in the following studies we consider potential differences in expression kinematics between posed, spontaneous, and communicative contexts.

The following collection of studies aims to first investigate whether speed differs as a function of emotion for posed, spontaneous, and communicative facial expressions (Experiments 1a–1c and 2) and thus establish whether kinematic cues, specifically speed, are indicative of an individual’s emotional state. Second, we aim to investigate whether manipulating speed and spatial cues impact on emotion recognition judgments (Experiments 3 and 4), and thus establish whether both kinematic, as well as spatial, cues are influential in observers’ judgments of emotion. To give a brief overview, Experiment 1 identifies (*N* = 47) and validates (*N* = 27) a new set of contemporary emotion induction videos that elicit discrete emotions of happiness, anger and sadness. Subsequently Experiment 1c (*N* = 42) demonstrates that (a) the speed of face movement differs as a function of emotion, (b) some face actions (e.g., mouth and eyebrow widening) are better than others (e.g., nose lengthening) with respect to differentiating emotions and, (c) while the general pattern of emotion-related speed differences is comparable for spontaneous and posed expressions, there are some differences in the utility of particular face actions. Experiment 2 (*N* = 67) comprises a partial replication of Experiment 1: that is, in an independent sample, we demonstrate differences in the kinematics of posed happy, angry and sad expressions. Experiment 2 also goes beyond this to show differences in speed between happy, angry, and sad for communicative expressions wherein participants are instructed to express emotion in spoken verbal utterances. Finally, in Experiments 3 (*N* = 29) and 4 (*N* = 41), we demonstrate that the emotion-related speed differences observed in Experiments 1 and 2 are influential cues that are used, alongside spatial cues, in emotion recognition. That is, we demonstrate that manipulating the speed of emotional expressions causally impacts on the accuracy of emotion recognition.

## Experiment 1a

An important consideration in the investigation of spontaneous expressions is the method of emotion-induction. Compared to other methods (e.g., pictures; Kuijsters et al., 2016; music; Krumhansl, 2002; and autobiographical recall; Prkachin et al., 1999), video induction appears to be one of the most successful means of inducing emotional states (see Kuijsters et al., 2016; Schaefer et al., 2010; Westermann et al., 1996). However, given that some of the most well-known video-sets for emotion induction were developed in the 80s and 90s (Gross & Levenson, 1995; McHugo et al., 1982), content may be outdated, and more contemporary videos may be more effective. Furthermore, many existing video-sets comprise short clips taken from full-length films, from which much of the necessary film context required to understand the clip is missing. Consequently, in Experiment 1a we selected short, self-contained, video clips that elicit emotions of happiness, anger, and sadness. We polled emotional responses to these videos in 47 healthy volunteers and demonstrated that emotion-induction was discrete: that is, the emotional response to each video clip was primarily driven by a single emotion (e.g., not a combination of happiness and sadness).

### Method

#### Participants

Forty-seven healthy volunteers (30 female, Aged 18–50 years) took part in an online task designed to select videos to induce specific emotional states in the observer. Participants had previously expressed willingness to take part in psychological research at the University of Birmingham and consent was received online, prior to task completion. Experimental procedures, for all experiments, were approved by the local research ethics committee (ERN 16-0281AP5) and were carried out in accordance with the principles of the revised Helsinki Declaration (World Medical Association, 2013).

#### Procedure

An online rating task included five videos selected to induce each of three target emotions (happy, angry, and sad; average length: 3.2 min; 15 videos in total). Videos were presented in a pseudorandom order (6 possible video orders); each was followed by rating scales. Participants were required to rate, in a random order, how happy, angry, sad, disgusted, surprised, and neutral they felt after each video (i.e., rating the target emotion plus all other nontarget emotions for each video). Participants also rated valence (positive/negative) and arousal levels following each video. Ratings were made on a 10-point Likert scale, whereby 1 indicated *not at all* and 10 indicated *very*. For valence ratings, 1 indicated *highly negative* and 10 indicated *highly positive*.

### Results

**Figure 1 fig1:**
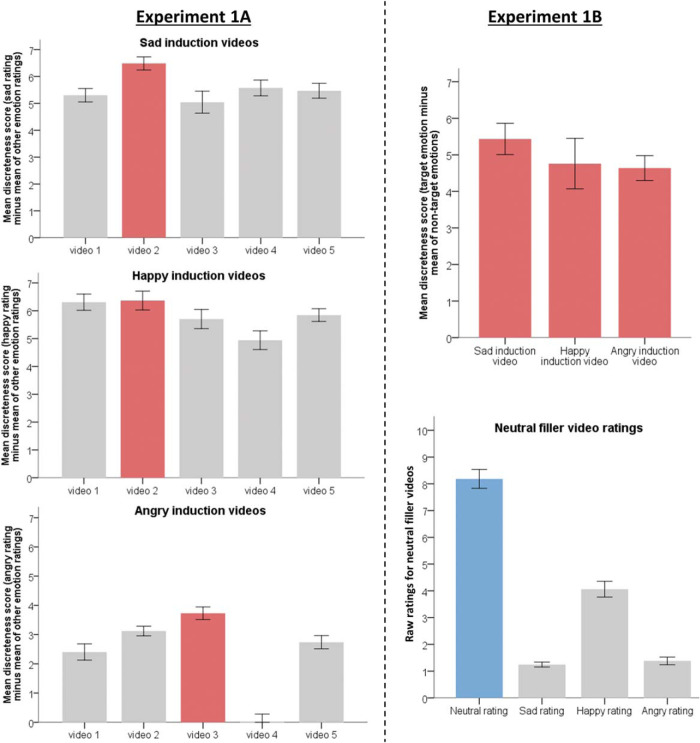
Emotion Rating Discreteness Scores From Experiments 1A and 1B for Target Emotions Sad, Happy, and Angry *Note.* (Left) Discreteness scores from Experiment 1A. Videos selected for use in Experiments 1B and 1C are highlighted in red. All discreteness scores are significantly different from zero except Video 4 for anger. (Right) Happy, angry, and sad videos, selected from Experiment 1A also resulted in discreteness scores that were significantly different from zero in Experiment 1B (top). Filler videos were successful: neutral ratings were significantly higher than ratings of happy, angry and sad (bottom). All error bars represent 1 *SEM*.

Mean valence ratings for happy videos [mean(*SEM*) = 7.88(0.15)] were significantly higher (more positive) than the scale midpoint [*t*(46) = 15.66, *p* < .001, *d* = 2.32]. Ratings for angry [mean(*SEM*) = 2.68(0.12)] and sad [mean(*SEM*) = 2.81(0.17)] videos were rated significantly lower (more negative) on the scale relative to the midpoint [anger: *t*(46) = −24.44, *p* < .001, *d* = 3.43], sad: [*t*(46) = −16.01, *p* < .001, *d* = 2.31]. Emotion-specific induction was successful (Figure 1, left panel): discreteness scores (target emotion rating minus mean rating of all nontarget emotions) for all videos (5 for each emotion), except one anger induction video, were significantly different from zero (all *p* < .001). The video providing the highest discreteness score for each target emotion was selected as a “winning” video and used in Experiment 1b (see the online supplemental materials Part A for details of the winning videos).[Fig-anchor fig1]

## Experiment 1b

### Method

Following the selection, in Experiment 1a, of the three winning videos, Experiment 1b validated these videos. That is, we demonstrated, in a sample of independent observers, that the same videos discretely elicited happy, angry and sad emotional responses.

#### Participants

A further 27 healthy student volunteers (21 females, Aged 18–35 years) were recruited via the University of Birmingham Research Participation Scheme in order to validate the final, chosen set of emotion induction videos. They gave written informed consent to take part and were reimbursed with a course credit or monetary incentive for their time. To achieve equivalent effect sizes to those achieved in Experiment 1a, power of 0.95 and error probability of 0.01, a priori power analysis revealed a minimum required sample size of six participants (G*Power Version 3.0.10; Faul et al., 2007). An increased sample size was chosen, on the basis that increasing the sample size improves the precision of effect size estimation in a replication sample (Asendorpf et al., 2013; Maxwell et al., 2008).

#### Procedure

Participants completed a video rating task of the shortlisted videos from Experiment 1a (see above for description of rating scales). They were seated at a computer, occluded from the view of the experimenter. The task involved watching the three winning video clips (average length: 3.0 min; see the online supplemental materials Part A) from Experiment 1a. Video order was pseudorandomized between participants. Between films, participants viewed a short neutral “filler” clip, to reset their mood to neutral and ensure no carryover of emotion induction from one condition to the next, on themes including pottery-making, gardening, and origami. Participants rated each video as in Experiment 1a. The experiment finished with a neutral video to ensure mood returned to neutral.

### Results

Valence ratings for neutral filler Videos 1 [mean(*SEM*) = 5.48(0.17)], 2 [mean(*SEM*) = 5.59(0.19)], and 3 [mean(*SEM*) = 5.11(0.19)] were not significantly different from the midpoint (the most “neutral” valence) on the scale (all *p* > .05). In line with Experiment 1a, valence ratings for the happy video [mean(*SEM*) = 7.59(0.42)] were significantly higher (more positive) than the midpoint [*t*(26) = 4.93, *p* < .001, *d* = 0.73], while those for the angry [mean(*SEM*) = 2.37(0.23)] and sad [mean(*SEM*) = 2.41(0.34)] videos were rated significantly lower (more negative) on the scale relative to the midpoint [anger: *t*(26) = −13.40, *p* < .001, *d* = 1.98; sad: *t*(26) = −9.02, *p* < .001, *d* = 1.33].

Following neutral filler videos, which were included to avoid the carryover of emotion induction from one condition to the next, participants were more likely to rate themselves as feeling neutral than happy, angry, and sad (all *p* < .001; see Figure 1, right panel, bottom). As in Experiment 1a, emotion induction was successful (see Figure 1, right panel, top); emotion rating discreteness scores (target emotion rating minus mean of nontarget emotions) for happy, angry, and sad videos were significantly different from zero (all *p* < .001).

## Experiment 1c

Following the selection and validation of videos, in Experiments 1a and 1b respectively, Experiment 1c measured (a) the kinematics of facial emotional expressions that were elicited by the videos, and (b) the kinematics of posed expressions of happy, angry, and sad emotions. We tested whether kinematics differed as a function of emotion and whether emotion-related speed differences were comparable across posed and spontaneous conditions. Building on evidence from the body movement literature, we hypothesized that, compared to sadness, anger and happiness would be associated with faster facial movements. To summarize our findings: in line with our hypothesis, we observed higher speed face movements for anger and happiness compared to sadness. Although this general pattern was true for both posed and spontaneous expressions, there were differences between these conditions in the particular face action that differentiated the emotions: eyebrow and mouth movements significantly differentiated posed emotions, however, spontaneous expressions of happiness, anger and sadness were differentiated by eyebrow movements alone.

### Method

#### Participants

Forty-two healthy student volunteers (39 female, Aged 18–35 years), recruited via the University of Birmingham Research Participation Scheme, gave written informed consent to take part and were reimbursed with a course credit or monetary incentive for their time. None of these participants had taken part in Experiments 1a or 1b. Seven further participants were excluded from analyses either due to poor registration with the facial tracking system (*N* = 4) or because of missing data (*N* = 3).

#### Procedure

Participants were seated with their head at the center of a polystyrene frame, positioned 80 cm from the computer monitor (21.5-in. iiyama display) and 1 m from a tripod-mounted video camcorder (Sony Handycam HDR-CX240E). Participants’ facial movements were recorded during two conditions (in the following set order):

1 Spontaneous—wherein participants watched the target emotion-induction videos selected and validated in Experiments 1a and 1b.
2 Posed—wherein participants posed the three target emotional expressions following the instruction to move from neutral, to peak expression, and back to neutral upon detecting a fixation cross with a coincident beep (9-s duration). See the online supplemental materials Part B for expression production instructions for both spontaneous and posed conditions.

The order of emotions was counterbalanced for spontaneous and posed conditions, while the order of recording condition, spontaneous followed by posed, was the same for all participants. To reset mood to neutral, emotion induction videos were interspersed with neutral filler videos. Following each video, for the spontaneous condition, participants completed the same ratings as described in Experiments 1a and 1b. Recordings for the spontaneous condition were cropped to the same 10-s scene rated, across all participants, as the most emotionally intense scene for each target emotion.

**Figure 2 fig2:**
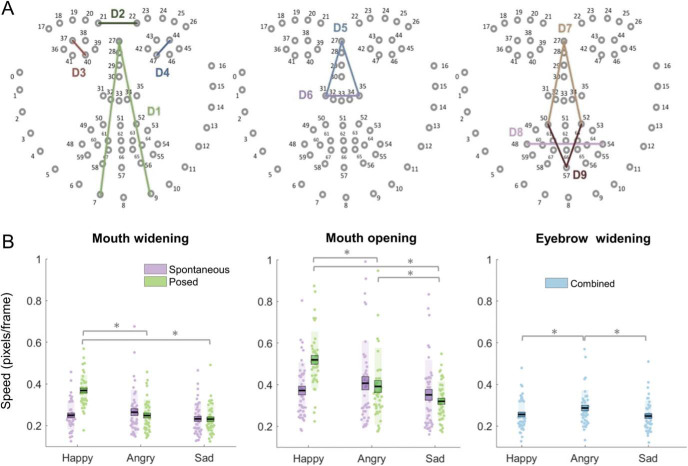
(A) Sixty-Eight Facial Landmarks Tracked by the Open-Source Facial Tracking Software OpenFace With Nine Face Distances Shown and (B) Plots From Experiment 1C of Mean Absolute Speed *Note.* (A) Face actions for Experiment 1C and 2 include eyebrow widening (labeled D2), nose lengthen (labeled D5 + D6), lip raise (labeled D7), mouth widen (labeled D8) and mouth open (labeled D9). (B) Plots from Experiment 1C are for each target emotion for mouth widening and mouth opening (purple = spontaneous, green = posed), and eyebrow widening collapsed across production condition. The asterisk denotes significance at *p* < .05, following Bonferroni alpha correction applied to each comparison. For each condition, data points indicate individual participants. The thick black horizontal line represents the sample mean, the surrounding box represents 1 *SEM* and the shaded region represents 1 *SD*.

Data analysis followed a novel pipeline, whereby recordings for each emotion, for posed and spontaneous conditions (6 videos per participant), were fed into the open-source software OpenFace (Baltrusaitis et al., 2018), which identifies locations, in pixels, of 68 2D facial landmarks (Figure 2A), sampled at a rate of 25 Hz. In order to address one of our key aims to quantify movement speed across different regions of the face, nine facial “distances” were calculated, following the procedure outlined in Zane et al. (2019). Specifically, key points on the face were identified and distances between these key points, corresponding to facial movements indicative of emotional expressions (Ekman & Friesen, 1977), were calculated as the square root of the sum of the squared differentials of the x and y coordinates of each key point.[Fig-anchor fig2]

For each face distance we calculated the Euclidian distance (radius) between key points for each successive frame according to Equation 1:

$$distance=\sqrt{{\left(x_{1}-x_{2}\right)}^{2}+{\left(y_{1}-y_{2}\right)}^{2}}.$$ 1

Face distances were then summed to create five face “actions,” as described in Zane et al. (2019), including inner eyebrow widening, nose lengthening, lip raising, mouth widening and mouth opening (details are presented in Table 1). For example, the eyebrow face action consisted of only distance D2 which represents furrowing and raising of the eyebrows. The face action for nose lengthening comprised the sum of D5 and D6 and thus represented average frame-to-frame changes in the distance from the top of the nose to the bottom of the left and right nostrils, reflecting a nose-wrinkling action often observed in expressions of disgust. Speed was represented as the average change in distance between relevant points on the face for each face action across each video clip. More specifically, speed was calculated as the differential of the face action vector (see Table 1) and represented as absolute values of each face action collapsed across all movements within a given time window. Thus, this is not the onset or number of frames taken to reach peak expressions, but rather absolute mean speed (pixels/frame) of movement observed within the recording window. Speed vectors were low pass filtered at 10 Hz to capture human movement and exclude noise associated with the MATLAB diff function (see MathWorks, n.d. for a discussion of this issue), and absolute speed was averaged, for each action, across each video. While in the Zane et al. (2019) study, a similar analysis procedure was applied to 3D coordinate data, it is important to note that here we instructed participants to keep their head within a polystyrene frame in line with a reference point and to use movement of the internal features of the face - as opposed to head movements—when making expressions, thus minimizing movement in the z plane. Correspondingly we analyze the data using a 2D coordinate system. Note that speed is reported in pixels/frame. For an average pupillary distance of 62 mm, recorded at a camera distance of 1 m, a reported movement of 0.5 pixels per frame movement is equivalent to ∼10 mm per second on the screen.[Table-anchor tbl1]

#### Statistical Analyses

In order to meet parametric criteria, speed data were subjected to a log-transformation (log_10_). To investigate whether kinematics differed as a function of emotion, spontaneous/posed expressions and face action, we conducted a repeated-measures analysis of variance (RM-ANOVA) with within-subjects factors condition (posed, spontaneous), emotion (happy, angry, sad) and action (eyebrow widen, nose lengthen, lip raise, mouth open, mouth widen). Where the assumption of sphericity was violated, Greenhouse-Geisser corrected values will be reported.

We predicted a main effect of emotion whereby kinematics differ across emotions. In addition, to reveal whether these kinematic effects differ across regions of the face as well as whether these are consistent across facial expression conditions (posed/spontaneous), we explored main effects of face action and condition, and interactions between these factors.

### Results

Replicating Experiments 1a and 1b, emotion induction was successful: emotion rating discreteness scores for each video were significantly greater than zero (all *p* < .001). In line with our prediction, the RM-ANOVA revealed a significant main effect of emotion [*F*(2, 82) = 14.14, *p* < .001, η_*p*_^2^ = .26]. Happy expressions had the highest speed, sad expressions were the slowest and angry expressions were intermediate (Table 2). A main effect of action was also observed [*F*(2.61,106.98) = 119.12, *p* < .001, η_*p*_^2^ = .74]: lip raise actions were fastest, mouth widen and eyebrow widen were slowest, and mouth open and nose lengthen were intermediate. There was no main effect of condition (*p* = .76). An Action × Emotion interaction indicated that the speed of eyebrow widening [*F*(2, 82) = 28.62, *p* < .001, η_*p*_^2^ = .41], mouth widening [*F*(2, 82) = 44.79, *p* < .001, η_*p*_^2^ = .52], and mouth opening [*F*(2, 82) = 27.01, *p* < .001, η_*p*_^2^ = .40], but not lip raising (*p* = .38) or nose lengthening (*p* = .08), differed as a function of emotion. Speed of eyebrow widening was highest for angry, lowest for sad and happy, while speed for mouth movements were highest for happy, followed by angry and lowest for sad. However, a significant interaction between condition, emotion, and action was also observed [*F*(4.74, 194.26) = 17.71, *p* < .001, η_*p*_^2^ = .30].[Table-anchor tbl2]

Separate RM-ANOVAs, for each action, revealed Condition × Emotion interactions for mouth opening [*F*(2, 82) = 18.17, *p* < .001, η_*p*_^2^ = .31] and mouth widening [*F*(2, 82) = 24.83, *p* < .001, η_*p*_^2^ = .38] only (*p* > .05 for all other actions). Bonferroni-corrected post hoc *t* tests revealed speed of both mouth opening and widening, during the posed condition, to be highest for happy expressions when compared to both angry (mouth widening [*t*(41) = 10.96, *p* < .001]; mouth opening [*t*(41) = 5.59, *p* < .001]) and sad (mouth widening [*t*(41) = 11.45, *p* < .001]; mouth opening [*t*(41) = 2.95, *p* < .001]). For mouth opening, angry expressions also elicited higher speed of movement compared to sad [*t*(41) = 2.95, *p* < .017]. No such differences were observed for spontaneous expression production (all *p*s > .05). See Figure 2B for a visual representation of the results.

### Interim Discussion 1

Experiments 1a and 1b identified and validated a new set of emotion-induction videos for the induction of three target emotions: happy, angry, and sad. Experiment 1c quantified whether emotional state impacts upon the movement kinematics of different face actions, and whether this differs between posed and spontaneous expressions. First, based on self-reported emotion ratings, the induction protocol employed was successful and showed significant, and discrete, emotion induction for happy, angry, and sad across three independent samples (Experiments 1a-1c). Second, data demonstrate that face movement kinematics provide important cues about emotional states. In line with the whole-body literature (Barliya et al., 2013; Michalak et al., 2009; Pollick et al., 2001; Sawada et al., 2003), anger and happiness were associated with high speed face movements, whereas sadness was characterized by low speed movements. Furthermore, we demonstrated that the speed of face actions differ in the extent to which they differentiate emotional states: eyebrow widening, mouth widening and mouth opening successfully differentiated emotions, whereas lip raising and nose lengthening did not. Finally, we also observed significant differences between posed and spontaneous conditions: whereas both eyebrow and mouth movements significantly differentiated posed emotions this was not the case for spontaneous expressions, which could only be differentiated by eyebrow movements.

## Experiment 2

Given the popularity of posed expressions in the facial emotion literature, in Experiment 2 we sought to replicate, in an independent sample, results from Experiment 1c. Thus, we predicted significantly faster face movements for happy and angry compared to sad posed expressions, and we predicted that both eyebrow and mouth would be particularly informative in discriminating posed expressions. Alongside this, we sought to design a novel measure of communicative, everyday facial emotion expression, where we examined facial movement kinematics during emotional spoken utterances. As noted above, although differences in recognition accuracy between communicative and noncommunicative expressions have been observed (Brewer et al., 2016), studies have not mapped expression kinematics in communicative and noncommunicative contexts. Indeed, only a handful of studies have considered facial emotion expressions during communicative spoken utterances (Graf et al., 2002; Hill et al., 2005; Kessous et al., 2010; Pelachaud et al., 1996). The analysis of facial emotion expression during spoken utterances is advantageous for a number of reasons. First, it provides a condition akin to much of our everyday social interaction, whereby facial expression is paired with speech. Second, this condition is well-matched to the abovementioned posed condition whereby the experimental setup is identical and participants are instructed to convey the same emotional states of happy, angry, and sad. Finally, it provides a comparison condition between the artificial context of posing caricature-style expressions (as used in the majority of dynamic face stimuli databases) and the more naturally evolving facial expressions that accompany emotional spoken utterances.

To precis our findings: in line with our predictions, we observed that both eyebrow and mouth actions discriminated posed expressions and, across all face actions, movements were faster for happy and angry, compared to sad. Thus, Experiment 2 partially replicates Experiment 1c. Although this general pattern was true for both posed and communicative (spoken) expressions, there were some differences between these conditions: specifically, the relationship between speed and emotion for eyebrow widening and lip raise movements differed as a function of (posed/spoken) condition.

### Method

#### Participants

Sixty-seven healthy student volunteers (56 female; *M*_age_ = 20.60), recruited via the University of Birmingham Research Participation Scheme, gave written informed consent to take part and were reimbursed with a course credit or monetary incentive for their time. Three further participants were excluded from analyses either due to poor registration with the facial tracking system (*N* = 2) or due to missing data (*N* = 1). None of the participants had taken part in Experiment 1. Based on the effect size achieved in Experiment 1c of 0.26 for the main effect of emotion on movement speed, with power of 0.95 and at an error probability of 0.01, a minimum required sample size of 34 participants was calculated (GLIMMPSE, Version 2; Kreidler et al., 2013). An increased sample size was chosen in the current experiment, on the basis that increasing the sample size should improve the precision of effect size estimation in a replication sample (Asendorpf et al., 2013; Maxwell et al., 2008).

#### Procedure

Participants were seated with their head at the center of a polystyrene frame, positioned 1 m from a tripod-mounted video camcorder (Sony Handycam HDR-CX240E). Participants’ facial movements were recorded during two conditions (in the following set order):

1 Spoken/communicative—wherein participants were instructed to verbalize a neutral sentence (“My name is John and I’m a scientist”) directly toward the camcorder; first in a neutral emotional state, followed by the three target emotional states (angry, happy, and sad; in a counterbalanced order). See the online supplemental materials Part C for instructions. Participants were provided with an example of a neutral, surprised and disgusted emotional expressive utterance (see the online supplemental materials Part D) and asked to practice each emotion to ensure they understood the procedure. They were encouraged to be as expressive as possible in order that someone could guess which emotion they were expressing.
2 Posed—wherein participants posed the three target emotional expressions (in a counterbalanced order) following the instruction to move from neutral, to peak expression, and back to neutral upon detecting a fixation cross with a coincident beep (9-s duration). Posed expression instructions remained the same as in Experiment 1c.

#### Statistical Analyses

Data analysis followed the same pipeline employed in Experiment 1c, whereby recordings for each emotion, for posed and spoken conditions (6 videos per participant), were fed into the open-source software OpenFace (Baltrusaitis et al., 2018) to recover facial landmarks. Across nine key face distances, five face actions were derived using the Zane et al. (2019) method and Equation 1 described in Experiment 1c. Speed was calculated as the differential of the action vector and represented as absolute values for each face action collapsed across all movements within a given time window. Thus, our dependent variable represents absolute mean speed (pixels/frame) of movement observed within the recording window. Speed vectors were low pass filtered at 10 Hz to capture human movement and exclude noise associated with the MATLAB diff function, and absolute speed was averaged, for each action, across each video.

In order to meet parametric criteria, speed data were subjected to a log-transformation (log_10_). To assess our main questions of whether facial movement speed differs as a function of emotion, as well as across different regions of the face and different emotion expression conditions (posed/spoken) we ran an RM-ANOVA with within-subjects factors condition (spoken, posed), emotion (happy, angry, sad), and action (eyebrow widen, nose lengthen, lip raise, mouth open, mouth widen). Where the assumption of sphericity was violated, Greenhouse-Geisser corrected values are reported.

### Results

The RM-ANOVA revealed a significant main effect of emotion [*F*(2, 132) = 39.02 *p* < .001, η_*p*_^2^ = .37]. Supporting results from Experiment 1c, happy and angry expressions were the fastest and sad expressions were the slowest. A main effect of face action was also observed [*F*(2.79,183.94) = 421.50, *p* < .001, η_*p*_^2^ = .86]: eyebrow widen actions were slowest, lip raise and mouth open were the fastest, while mouth widen and nose lengthen were intermediate. Utilizing the new spoken form of facial emotion expression, intuitively in this dataset, there was a main effect of condition [*F*(1, 66) = 1181.01, *p* < .001, η_*p*_^2^ = .95]; whereby facial movement speed was higher during spoken compared to posed emotion expressions. Table 3 displays descriptive statistics.[Table-anchor tbl3]

**Figure 3 fig3:**
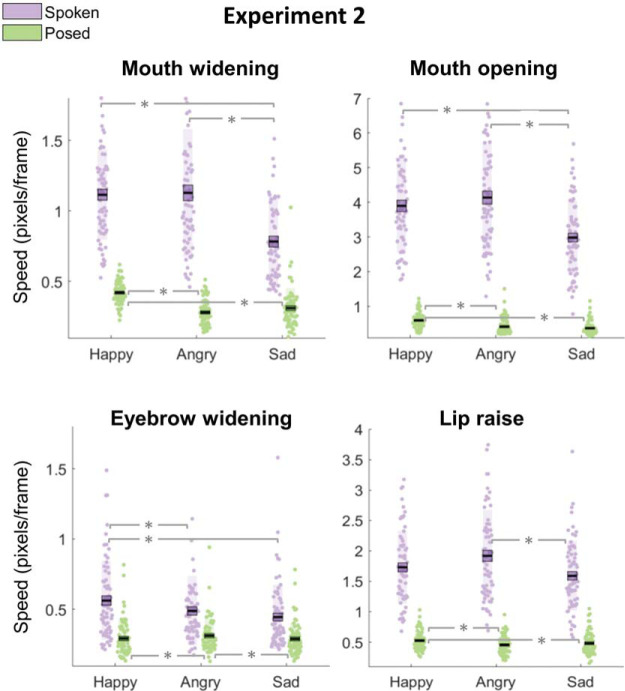
Plots From Experiment 2 of Mean Absolute Speed for Each Target Emotion for Mouth Widening, Mouth Opening, Eyebrow Widening and Lip Raise Across Conditions (Purple = Spoken, Green = Posed) *Note.* The asterisk denotes significance at *p* < .05, following Bonferroni alpha correction applied to each comparison. For each condition, data points indicate individual participants. The thick black horizontal line represents the sample mean, the surrounding box represents 1 *SEM* and the shaded region represents 1 *SD*.

We observed a Face Action × Condition × Emotion [*F*(5.47, 360.70) = 24.68, *p* < .001, η_*p*_^2^ = .27] interaction. Follow-up RM-ANOVAs demonstrated (a) a significant interaction between emotion and face action for both posing [*F*(5.18, 341.52) = 37.65, *p* < .001, η_*p*_^2^ = .36] and spoken [*F*(5.52, 364.20) = 13.45, *p* < .001, η_*p*_^2^ = .17] conditions, (b) a significant Emotion × Condition interaction for all face areas (all *p*s < .05; though the interaction for nose lengthening was marginally significant, *p* = .04), and (c) a significant Face Action × Condition interaction for happy [*F*(2.94, 193.76) = 214.92, *p* < .001, η_*p*_^2^ = .77], angry [*F*(2.98, 196.62) = 393.56, *p* < .001, η_*p*_^2^ = .86], and sad [*F*(2.88, 189.95) = 255.84, *p* < .001, η_*p*_^2^ = .80]. However, the way in which speed was modulated across face regions differed as a function of condition and emotion (Figure 3 displays a visual depiction of results). More specifically, in line with our results from Experiment 1c, for posed emotions, all mouth movements were numerically fastest for happy, slowest for sad, and intermediate for angry expressions. The remaining results are presented following Bonferroni alpha correction. Post hoc *t* tests revealed that for mouth widening, mouth opening, and lip raising, angry and sad were comparably slow (all *p*s > .05, Bayes factor (BF_01_; mouth widen) = 1.45, BF_01_(mouth open) = 3.15, BF_01_(lip raise) = 4.20) and happy was significantly faster than both angry (all *t*s ≥ 4.24, *p* < .05) and sad (all *t*s ≥ 3.00, *p* < .05). Bayes factors (BF01) here provide a ratio of likelihood for the observed data under the null compared to the alternative hypothesis. Values of 3–10 and 1–3 will be taken as moderate and anecdotal evidence for the null hypothesis respectively (Lee & Wagenmakers, 2014).[Fig-anchor fig3]

Spoken sentences differed from this pattern. Although it was also true that for spoken sentences mouth opening and widening movements were significantly faster for happy versus sad (all *t*s ≥ 8.00, *p* < .05), it was also consistently the case that happy and angry were comparably fast (all *p*s > .05, BF_01_(mouth widen) = 6.57, BF_01_(mouth open) = 2.82) and sad was slower than angry (all *t*s ≥ 9.69, *p* < .05). With respect to lip raise actions, Bayesian statistics provided anecdotal evidence that angry and happy expressions were comparably fast (*p* > .05, BF_01_ = 0.85), angry was faster than sad, *t*(66) = 4.53, *p* < .001, but happy was not faster than sad (*p* > .05, BF_01_ = 1.10). In other words, for both posed and spoken expressions happy mouth movements were fastest, sad was slowest and angry was intermediate. However, for posed expressions, differences between the emotions were driven by happy being faster than sad and angry; for spoken sentences, differences were driven by sad been slower than happy and angry.

For eyebrow widening we saw a different pattern wherein, for posed expressions, replicating the result from Experiment 1c, speed was comparable for happy and sad (*p* > .05, BF_01_ = 7.45) and faster for angry (all *t*s ≥ 3.00, *p* < .05). Whereas for spoken, we observed anecdotal evidence that speed was comparable for angry and sad (*p* > .05, BF_01_ = 0.66) and faster for happy (all *t*s ≥ 2.68, *p* < .05).

### Interim Discussion 2

In Experiment 2, we demonstrated a replication, in an independent sample, of the effects observed in Experiment 1c for posed facial emotion expressions. Movement kinematics of eyebrow and mouth movements significantly differed between emotions. While eyebrow widening movements were significantly faster during expressions of anger compared to happiness and sadness, mouth movements were significantly faster for happy compared to angry and sad expressions. We also introduced a new measure of dynamic, communicative facial emotion expression that elucidates the kinematics of expression during verbal utterances, and which is highly relevant to our everyday social interactions. In line with intuition, facial movement during spoken utterances is faster than the corresponding posed expressions. Nevertheless, there are both similarities and differences between posed and spoken expressions: as when posing a facial expression, when attempting to convey happiness or anger via a verbal utterance, individuals use high-speed mouth widening and opening movements; in contrast, sadness is characterized by lower speed mouth movements. Unlike facial emotion expressions that do not contain speech (i.e., posed or spontaneous facial emotion), eyebrow movements during emotional spoken utterances are faster for happy compared to angry and sad expressions, and angry lip raise actions are faster than those for happy and sad utterances.

The data presented thus far provide evidence that humans exhibit differences in facial movement speed across emotional expressions. Despite some differences across emotional expression contexts (i.e., posed, spontaneous or communicative verbal utterances), across different face actions, happy and angry expressions are consistently typified by high-speed movement compared to the low speed movement observed for expressions of sadness. Thus, it appears that as well as the presence of specific face actions (i.e., static cues such as the position of the eyebrows and mouth; Ekman & Friesen, 1977), the movement speed of such actions (i.e., kinematic information) may provide an additional cue to guide social interaction partners in understanding one’s current emotional state.

## Experiment 3

Experiments 1 and 2 demonstrate that kinematic information differs as a function of emotion. Since the speed of face movement differentiates emotions, observers could use this information as a cue to help them to label emotional expressions. At present, however, it is unclear whether this is the case. Although previous studies have demonstrated that recognition is improved for dynamic (e.g., videos), compared to static, displays of facial emotion (Ambadar et al., 2005; Bould & Morris, 2008; Bould et al., 2008). It is currently unclear as to whether the dynamic-over-static advantage is driven by temporal or kinematic cues. In other words, although kinematic information would be a useful cue, it is not clear that observers actually use it in the process of emotion recognition.

It has previously been demonstrated that disrupting the temporal order of action units within a sequence negatively impacts emotion recognition (Delis et al., 2016; Jack et al., 2014; Jack & Schyns, 2015). Thus, temporal information accounts for at least some of the dynamic-over-static advantage. A small number of studies have tried to ascertain whether disrupting kinematic information, also influences emotion recognition, however, findings are mixed. Kamachi et al. (2013) demonstrate that happy expression recognition is improved when expressions are increased in speed, while recognition of sad expressions is impaired. However, in many such paradigms (Kamachi et al., 2013; see also Fayolle & Droit-Volet, 2014; Sato & Yoshikawa, 2004), speed is artificially altered by morphing between static neutral and static facial emotion expressions to create the illusion of a dynamic, moving face. Such artificial stimuli may not truly capture the temporal sequence or kinematics of emotional expressions. Countering this issue, Hill et al. (2005) used infrared markers to record facial movement directly from real human actors who expressed emotion through spoken utterances. Kinematic and spatial manipulations were then made directly from these movement patterns and presented on synthetic faces—thus creating stimuli with nonartificial movement patterns. They investigated the impact on emotion intensity perception of manipulating both the spatial and kinematic content of the videos. In conflict with Kamachi et al.’s (2013) findings, Hill and colleagues (2005) observed an effect of spatial, but not kinematic, manipulation.

Moreover, an issue that may be adding noise to the literature is that studies where face kinematics have been manipulated often employ stimuli where features such as identity, gender, age and other observable demographic information can provide additional cues about the underlying emotional state (Calder & Young, 2005). The more additional cues there are, the less demand there is on kinematic information. Countering these issues, Pollick and colleagues (2003) presented expressions recorded from human actors as point light displays. These displays represent real-time movement of a specific number of points on the face, displayed as a series of moving dots with all other visual information (including identity cues) removed. To assess the contribution to emotion recognition of both spatial and kinematic information, Pollick and colleagues (2003) manipulated spatial and kinematic information in the point light displays. They observed that spatial, but not kinematic, manipulation influenced emotion recognition. Thus, suggesting that kinematic cues may not be used by observers. It is important, however, to note that Pollick and colleagues (2003) and Hill et al. (2005) exaggerated spatial and kinematic information in separate stimuli and investigated the effect of these manipulations in separate experiments. Thus, the contributions of spatial and kinematic information could not be compared within-participant and the alternative explanation, that the sample recruited for the kinematic experiment were generally less susceptible to any sort of stimulus manipulation, cannot be ruled out. To summarize, existing literature suggests that spatial and temporal cues influence emotion recognition. However, studies are mixed with respect to the use of kinematic cues. To address methodological issues, this field needs studies that use point light stimuli that represent the natural kinematics of emotion transitions (i.e., not motion morphs) and paradigms wherein the influence of spatial and kinematic manipulation can be compared within-participant and with these factors crossed within the same stimuli.

The following two experiments (Experiment 3: discovery [*N* = 29] and Experiment 4: replication [*N* = 41]) addressed the question of whether kinematic, in addition to spatial, information is used in facial emotion expression recognition. We present an emotion recognition task that removes identity information through the use of point light displays. These were created from recordings of the faces of actors who were asked to convey happy, angry and sad emotional states while verbalizing a neutral sentence. We then created both a spatial manipulation (increasing and decreasing spatial movement extent) and a kinematic manipulation (increasing and decreasing movement speed) from the same original stimuli; crossing these factors when creating task stimuli. Participants were required to rate (on a scale from 0 to 10) the extent to which they believed each stimulus depicted each of our target emotions (happy, angry, and sad). This (multiple-emotion rating) removed the requirement for participants to make single fixed-choice emotion responses, leaving them free to rate any intensity on each scale from *Not at all happy/angry/sad* to *Very happy/angry/sad.* This also facilitates an examination of the impact of manipulating spatial and kinematic features on subtle emotion confusions. Emotion recognition scores were calculated as the rating given for the target emotion minus the mean of the two nontarget emotions.

In line with previous literature on the role of spatial cues in facial emotion perception (Calder et al., 1997, 2000; Ekman & Friesen, 1977; Hill et al., 2005; Pollick et al., 2003), we predicted a main effect of spatial manipulation, whereby spatially exaggerating emotional facial expressions would lead to higher emotion recognition scores. Regarding the kinematic manipulation, based on our findings from Experiments 1 and 2—whereby fast movement was associated with happy and angry expressions and sad was associated with slow expression movement—we predicted a significant Emotion × Kinematic interaction, whereby increasing speed of facial emotion expressions of happy and angry leads to increased emotion recognition accuracy, while decreasing speed of sad emotional expressions leads to increased recognition accuracy. We also explored emotion confusions that participants made as a result of kinematic manipulation. To precis our findings: we observed an influence of both spatial and kinematic manipulation. Furthermore, the influence of kinematic information was consistent with our predictions such that speeding up movement improved accuracy for happy and angry expressions, whereas slowing down movement improved accuracy for sad expressions.

### Method

#### Participants

Twenty-nine healthy student volunteers (26 female; *M*_age_ = 20.34), recruited via the University of Birmingham Research Participation Scheme, gave written informed consent to take part and were reimbursed with a course credit or monetary incentive for their time. Sample size was calculated based on the study by Edey and colleagues (2017) whereby similar manipulations of emotion and stimulus speed levels were made (albeit to whole-body point light displays). To achieve an equivalent effect size (η_*p*_^2^ of 0.23 in the main interaction of interest), power of 0.95 and error probability of 0.01, a minimum sample size of 22 participants is required (G*Power 3.0.10).

#### Materials and Stimuli

The computerized task was programmed using Cogent and Cogent Graphics (Wellcome Department of Imaging Neuroscience), run using MATLAB 2018a (MathWorks, Natick, MA), on a Stone (Stone Computers, Stafford, UK) computer and presented on a 21.5-in. iiyama display monitor (refresh rate: 60 Hz; resolution 1,280 × 1,024). Stimuli were created from videos (made using a Sony Handycam HDR-CX240E) of four actors (2 male, 2 female) instructed to hold their head still while expressing one of three target emotional states (happy, angry, and sad) via a spoken sentence (“My name is John and I’m a scientist”). Video recordings of each expression were fed into OpenFace from which the *x* and *y* coordinates of 68 facial landmarks on the face were extracted at 25 frames per second (FPS). To create the stimuli we then displayed these successive frames of coordinates as white dots at 25 FPS on a black background screen (using Cogent graphics for MATLAB) and saved these displays as video files to be presented during the computerized task. We will refer to these stimulus videos as point light display faces (PLFs). Stimulus videos of spoken sentence expressions displayed facial movement without sound, a method previously found to successfully convey the intended emotion (Hill et al., 2005).

The kinematic manipulation comprised three levels (K1: 50% original stimulus speed; K2: 100% original stimulus speed and K3: 150% original stimulus speed). When creating stimulus videos for K1, K2, and K3 we saved PLF frames at varying frame rates to create the illusion of varying facial movement speed. Videos for kinematic Level 2 (K2) were saved at the original 25 FPS (as described above), while videos for K1 and K3 kinematic levels were created by reducing and increasing the frame rate by 50% such that K1 was 12.5 FPS and K3 was 37.5 FPS. The stimuli varied in total length, however, length of display has previously been shown in both a point light display task of whole-body emotion movement (Edey et al., 2017) and dynamic facial morphing tasks (Hill et al., 2005; Kamachi et al., 2013) to have no impact on emotion recognition performance. For instance, Edey and colleagues demonstrated that their effects of interest were observed for both their original stimuli (point light displays that were not matched in overall length) and control point light displays where the length of video across speed manipulations was held constant by looping the faster videos such that they matched the length of the slower videos.

The spatial manipulation also comprised three levels (S1: 50% spatial movement; S2: 100% spatial movement and S3: 150% spatial movement). Stimulus videos for S2 were created by presenting *x* and *y* coordinates for each of the 68 facial landmarks for each frame as recorded by OpenFace. However, for stimulus videos at S1 and S3 spatial levels, the movement of each facial landmark from one frame to the next was reduced (x0.5 spatial distance moved for S1) or increased (x1.5 spatial distance moved for S3) to create the apparent reduction or increase in spatial exaggeration of the facial emotion expressions recorded. For each successive pair of frames we calculated the Euclidian distance (radius) according to Equation 2:

$$distance=\sqrt{{\left(x-x_{\text{previous}\;\text{frame}}\right)}^{2}+{\left(y-y_{\text{previous}\;\text{frame}}\right)}^{2}}$$ 2

and angle of direction of movement (θ) according to Equation 3:

$$\theta =\text{atan}\left(\frac{\left|y-y_{\text{previous}\;\text{frame}}\right|}{\left|x-x_{\text{previous}\;\text{frame}}\right|}\right).$$ 3

The new spatially manipulated location of the dot was given by Equation 4:

$$x_{\text{manipulated}}=x_{\text{manipulated}\;t-1}-\left(k\text{.radius}\right)\text{cos}\;\theta$$

$$y_{\text{manipulated}}=y_{\text{manipulated}\;t-1}-\left(k\text{.radius}\right)\text{sin}\;\theta$$ 4

where *k* = 0.5 for S1 and *k* = 1.5 for S3. That is, by applying the multiplication factor, *k*, to the radius, but not to θ the original trajectory of movement of each landmark was maintained while the spatial extent was reduced or increased. Beginning at the original starting location, this manipulation was then applied sequentially to each sample in the stimulus video, for each point on the face. Note that for the Cogent Graphics coordinate system the origin (0,0) is in the top left of the computer screen.

Stimuli were created from four actors expressing each target emotion (happy, angry and sad) for each kinematic manipulation level (K1, K2, and K3), at each level of spatial manipulation (S1, S2, and S3). This resulted in 108 video stimuli (average length of 2 s per video).

#### Procedure

**Figure 4 fig4:**
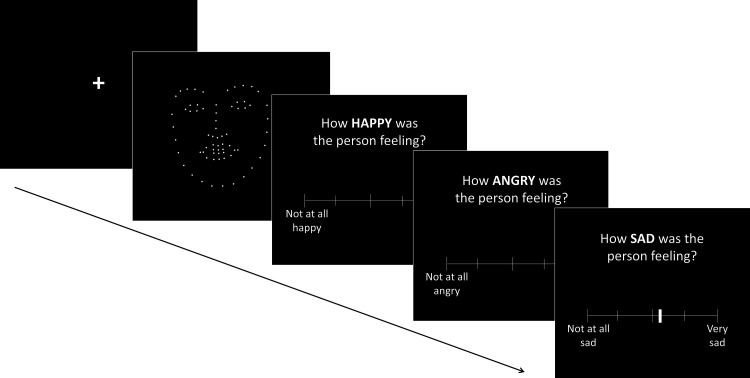
Example of One Trial in the Emotion Perception Task From Experiments 2 and 3 *Note.* Fixation cross display is presented for 500ms at the start of each trial. See online supplemental materials Part E for links to download example point light display face stimuli videos for angry, happy and sad.

Participants were seated 80 cm from the computer monitor, whereby PLF stimuli subtended an average visual angle of 7.51° along the vertical plane and 7.87° along the horizontal plane. This stimuli presentation corresponds to roughly the size of these facial features on an average human face. Participants performed an emotion perception task of roughly 40 min. Each trial in the experiment (Figure 4) began with the presentation of a stimulus video; a silent PLF video of one actor expressing one of three emotions via a spoken sentence, presented at one of three kinematic and three spatial levels. Following this, they were asked to rate how happy, angry, and sad each of the faces they were shown were feeling. Individuals moved their cursor along the scale (see Figure 4) to their chosen response, with one end representing *Not at all happy/angry/sad* and the opposite end representing *Very happy/angry/sad*. Participants were asked to rate the three emotions of happy, angry and sad, with scales for these presented on the screen in a random order, after each stimulus video. They were not required to make these three ratings sum to 10 and thus ratings were independent of one another. Participants first completed three practice trials, before completing 108 randomly ordered experimental trials across three blocks.[Fig-anchor fig4]

### Results

Emotion rating responses for each emotion scale on each trial were transformed into scores from 0 to 10 (with 0 representing a response of *Not at all* and 10 a response of *Very*). Emotion recognition scores were calculated as the target emotion rating minus the mean of the two nontarget emotion ratings. For example, for a trial on which a happy PLF was presented, the mean ratings of the two nontarget emotions (angry and sad) were subtracted from the target emotion, happy.

RM-ANOVA was used to analyze emotion recognition accuracy with within-subjects factors emotion (happy, angry, sad), kinematic manipulation (K1, K2, K3) and spatial manipulation (S1, S2, S3). This revealed a significant main effect of emotion [*F*(2, 56) = 10.50, *p* < .001, η_*p*_^2^ = .27] with recognition scores highest for happy [mean(*SEM*) = 4.65(0.34)], lowest for angry [mean(*SEM*) = 3.14(0.19)] and intermediate for sad expressions [mean(*SEM*) = 3.85(0.25)]. A main effect of spatial manipulation was observed [*F*(2, 56) = 183.68, *p* < .001, η_*p*_^2^ = .87]: S3 stimuli elicited highest recognition scores [mean(*SEM*) = 5.18(0.21)], followed by S2 [mean(*SEM*) = 4.11(0.24)] and then S1 [mean(*SEM*) = 2.34(0.18)]. However, this was qualified by a significant Emotion × Spatial interaction [*F*(4, 112) = 66.71, *p* < .001, η_*p*_^2^ = .70] indicating that for happy and angry videos, recognition scores were highest for high spatial exaggeration S3, followed by S2 and finally S1; whereas for sad videos low spatial exaggeration S1 elicited highest emotion recognition scores, while S2 and S3 were equally rated less accurately. Means are presented in Table 4. There was no main effect of kinematic manipulation (*p* = .266).[Table-anchor tbl4]

**Figure 5 fig5:**
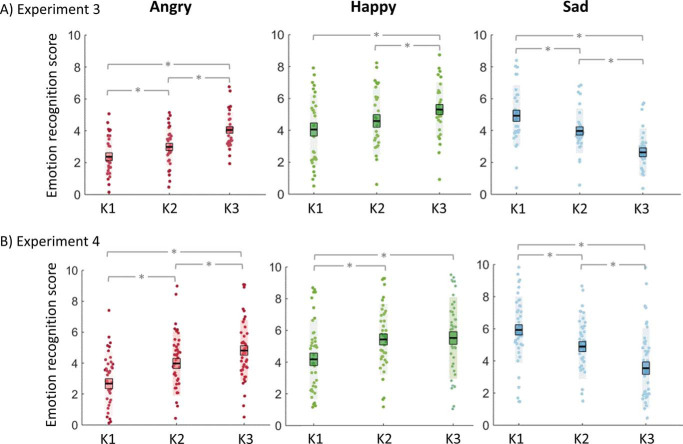
Plots of Emotion Recognition Task Results for Experiment 3 (A) and Experiment 4 (B) for Angry (Red), Happy (Green), and Sad (Blue) Facial Emotion Stimuli *Note.* Emotion recognition scores shown on the *y*-axis (emotion rating given to target emotion—mean emotion rating given to the two nontarget emotions) and scores are presented at each stimulus speed level from the slowest (K1) to the fastest (K3). The asterisk denotes significance at *p* < .05, following Bonferroni alpha correction applied to each comparison. For each condition, data points indicate individual participants. The thick black horizontal line represents the sample mean, the surrounding box represents 1 *SEM* and the shaded region represents 1 *SD*.

Crucially, in line with our first prediction, we found an Emotion × Kinematic interaction [*F*(4, 112) = 31.47, *p* < .001, η_*p*_^2^ = .53]. Follow-up RM-AMOVAs conducted separately for each emotion revealed a significant main effect of kinematic manipulation for happy [*F*(2, 56) = 8.91, *p* < .001, η_*p*_^2^ = .24], angry [*F*(2, 56) = 33.64, *p* < .001, η_*p*_^2^ = .55] and sad [*F*(2, 56) = 35.92, *p* < .001, η_*p*_^2^ = .56]. Figure 5 displays a visual depiction of this result. Emotion-specific recognition increased with increasing speed from K1 through K3 for both angry and happy stimuli; whereas for sad videos emotion-specific recognition increased as speed *decreased*, from K3 through K1.[Fig-anchor fig5]

#### Independence and Magnitude of Spatial and Kinematic Effects

To address the question of the independence of spatial and kinematic effects on accuracy, we explored Spatial × Kinematic and Emotion × Spatial × Kinematic interactions, neither of which were statistically significant (all *p*s > .05). Bayesian statistics demonstrated that we had very strong evidence to accept the null hypothesis that there was no interaction between spatial and kinematic (BF_01_ = 99) or emotion, spatial and kinematic factors (BF_01_ = 33). Moreover, we found that the significant Emotion × Kinematic interaction persisted across all three levels of spatial manipulation, when spatial information was highly degraded (S1 [*F*(4, 112) = 8.39, *p* < .001, η_*p*_^2^ = .09]), when spatial information was unaltered (S2 [*F*(4, 112) = 26.99, *p* < .001, η_*p*_^2^ = .49]) and when spatial information was exaggerated (S3 [*F*(4, 112) = 16.11, *p* < .001, η_*p*_^2^ = .37]). When considering only trials at spatial Level 2 (i.e., where spatial information was not manipulated to ensure that we remove any influence that spatial exaggeration has on kinematic exaggeration), emotion recognition accuracy still increases with increasing speed for both angry and happy stimuli; whereas emotion-specific recognition for sad videos increased as speed decreased (see the online supplemental materials Part F for descriptive statistics).

To quantify whether accuracy is more affected by kinematic or spatial manipulations, we ran two further analyses. First, examining the absolute change in accuracy from the highest to lowest spatial levels (S3–S1) at the kinematically unmanipulated (K2) level relative to the absolute change in accuracy from the highest to lowest kinematic levels (K3–K1) at the spatially unmanipulated (S2) level, revealed that spatial manipulation (at 100% [K2] speed) more greatly affected accuracy than kinematic manipulation (at 100% [S2] spatial extent) for happy [*t*(28) = 5.36, *p* < .001, *d* = 1.43] and angry stimuli [*t*(28) = 6.33, *p* < .001, *d* = 1.54]. However, for sad stimuli the direction of the effect was reversed such that kinematic manipulation more greatly affected accuracy than spatial manipulation [*t*(28) = −2.40, *p* = .023, *d* = 0.47]. Note, however, that this effect does not reach significance at a Bonferroni-corrected alpha cutoff of 0.0167 for the three *t* tests (happy, angry, and sad). This analysis suggests that whereas judgments of happiness and anger are more greatly influenced by spatial versus kinematic manipulation, this is not the case for judgments of sadness. For sadness there is a trend toward a greater effect of kinematic over spatial manipulation.

The second analysis took this one step further by investigating the effect of spatial manipulation while controlling for the speed of movement of facial features. Since speed and distance are intrinsically linked, our spatial manipulation also affects the speed of movement. That is, trials in the S1 condition are at 50% of spatial movement and thus facial features move 50% slower than normal (100% [K2] speed), whereas, trials in the S3 condition are at 150% of spatial movement and 50% faster than normal. Comparing S3 K1 and S1 K3 controls for speed because for both conditions speed is 75% of normal (100% [K2]) speed.^1^ Comparing accuracy for S3 K1 versus S1 K3 stimuli revealed that participants perform significantly better for S3 compared to S1 for both happy [mean S3(*SEM*) = 6.06(0.43), mean S1(*SEM*) = 2.78(0.43); *t*(28) = 7.16, *p* < .001, *d* = 1.42] and angry [mean S3(*SEM*) = 4.96(0.33), mean S1(*SEM*) = 1.28(0.29); *t*(28) = 9.93, *p* < .001, *d* = 2.20]. Thus, confirming that for angry and happy expressions the spatial manipulation affects accuracy even when kinematic differences are controlled for. Based on the significant emotion by spatial interaction in our main ANOVA, whereby sad expression accuracy is enhanced by reduced spatial exaggeration (S1 relative to S3), we would expect to find the opposite effect for sad expressions. However, if in line with our original hypothesis that spatial exaggeration may lead to increased recognition accuracy across emotions, we would alternatively expect to find that higher accuracy is observed at S3 compared to S1 when controlling for speed. We indeed found that sad accuracy increased from spatial Level 1 to 3, in the same direction as the effect observed for happy and angry [mean S3(*SEM*) = 4.85(0.37), mean S1(*SEM*) = 3.82(0.41); *t*(28) = 2.267, *p* = .031, *d* = 0.49]. Although again note that this effect is only trending toward significance at a Bonferroni-corrected alpha cutoff of 0.0167.

Together, these two analyses suggest that, while clear effects of kinematics can be seen across all emotions, recognition accuracy for happiness and anger is more greatly influenced by spatial versus kinematic manipulation. Consequently, for these emotions spatial effects on accuracy are observed even when the speed of the facial features is matched. For sadness, however, there is a trend toward a greater effect of kinematic over spatial manipulation. The omnibus ANOVA shows that spatial exaggeration (e.g., S3 vs. S1 at the K2 level) reduces accuracy for sad stimuli. However, when the speed of facial features is matched between conditions there is a trend toward greater accuracy for spatially exaggerated stimuli.

#### The Impact of Kinematics on Emotion Confusions

**Figure 6 fig6:**
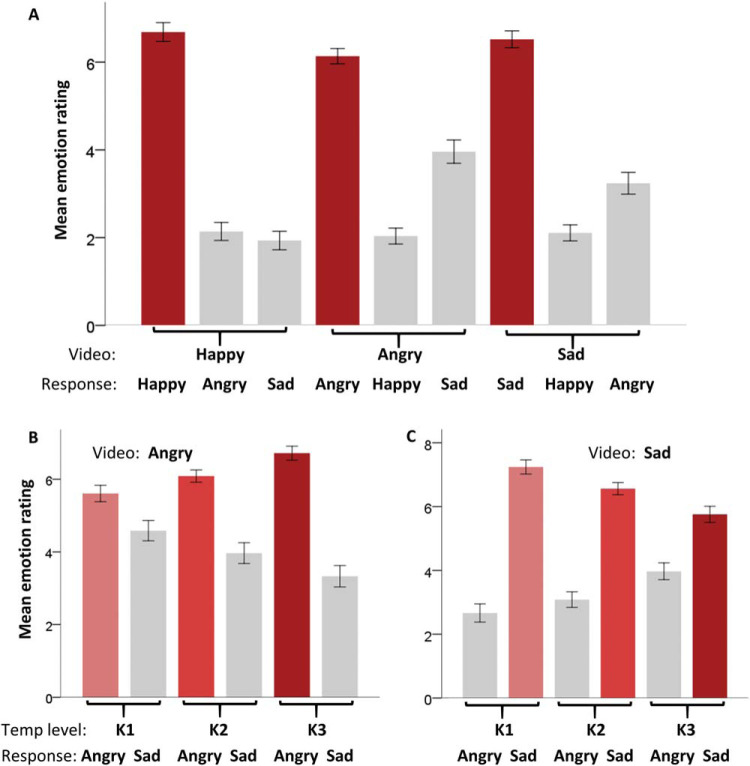
Plots From Experiment 3 *Note.* (A) Mean emotion ratings for happy, angry, and sad for each of the target emotion videos happy, angry, and sad (B). Mean emotion ratings given on angry and sad scales for both the angry (left) and sad (right) videos, for speed levels K1, K2, and K3. Demonstrating as angry videos increase in speed, sad ratings decrease, while as sad videos increase in speed, angry ratings increase. Error bars represent 1 *SEM*.

Our final exploratory question, as previously investigated with whole-body dynamic emotional stimuli (Atkinson et al., 2004; Dittrich et al., 1996), regards whether specific emotion confusions are made as PLF stimulus videos transition away from their typical speed. For example, here we refer to emotion confusions made when happy and angry videos decrease in speed and sad videos increase in speed. As presented in Figure 6A, happy, angry, and sad videos were consistently rated highest for the correct emotion (e.g., happy rating highest for happy videos). However, while happy videos were equally likely to be confused for angry or sad, it was found that both angry and sad videos were more likely to be confused with one another. Figure 6B and 6C demonstrates that as the speed of angry videos decreases they are rated as less angry [*F*(2, 56) = 25.62, *p* < .001, η_*p*_^2^ = .48] and are more likely to be confused for sad [*F*(2, 56) = 21.83, *p* < .001, η_*p*_^2^ = .44]. Conversely, as the speed of sad videos increases they are rated as less sad [*F*(2, 56) = 28.50, *p* < .001, η_*p*_^2^ = .50] and are more likely to be confused for angry [*F*(2, 56) = 36.76, *p* < .001, η_*p*_^2^ = .57]. No such differences were observed in happy ratings across different kinematic levels of angry or sad videos (all *p*s > .05).[Fig-anchor fig6]

In sum, in line with previous literature, we demonstrate that spatial features of facial expressions play an important role in facial emotion recognition. However, as predicted, we also demonstrate that kinematic features of facial movement (specifically movement speed) independently contribute to emotion recognition accuracy. This contribution can be distinguished from the influence of spatial information and indeed persists across all levels of spatial exaggeration/reduction and when spatial information is not manipulated. Specifically, we observed that increasing the speed of movement improved recognition of angry and happy expressions and impaired recognition of sad expressions; decreasing speed improved recognition of sad expressions and impaired recognition of angry and happy expressions. We also demonstrate that, despite an impact of both spatial and kinematic manipulations across all emotions, happy and angry expression accuracy may be more greatly impacted by spatial exaggeration, while sad expression accuracy more greatly impacted by kinematic manipulation. A possible explanation for this effect is that, as with anger and happiness, spatial exaggeration of sadness improves accuracy and the (opposite) effect observed in the omnibus ANOVA (reduced accuracy for S3 relative to S1) is due to the combination of (a) spatial exaggeration increasing speed and (b) kinematic information presiding over spatial information thus meaning that the increase in speed outweighs any benefit of spatial exaggeration. In Experiment 4 we sought to provide an independent replication of these effects of speed on emotion recognition.

## Experiment 4

### Method

#### Participants

A replication sample of 41 healthy student volunteers (32 female, *M*_age_ = 20.88), recruited via the University of Birmingham Research Participation Scheme, gave written informed consent to take part and were reimbursed with a course credit or monetary incentive for their time. We aimed to replicate the kinematic effects found in Experiment 3, while not manipulating spatial information. Considering the presence of our main interaction effect of interest in the previous experiment (partial eta squared of 0.53), and a priori power analyses that revealed a required sample size of 11 to replicate the interaction effect in an independent sample (alpha value of 0.01, power of 0.95; GLIMMPSE Version 2), an increased sample size was chosen in the current experiment on the basis that increasing the sample size should improve the precision of effect size estimation in a replication sample (Asendorpf et al., 2013; Maxwell et al., 2008).

#### Materials and Stimuli

Materials were identical to Experiment 3, while stimuli were a subset of those described in Experiment 3. These included the same PLF stimuli created from four actors (2 male, 2 female) instructed to express three target emotional states (happy, angry, and sad) via a spoken sentence (“My name is John and I’m a scientist”). In this version of the task, we used only stimuli from the kinematic manipulation (K1: 50% original stimulus speed; K2: 100% original stimulus speed and K3: 150% original stimulus speed), not the spatial manipulation. This resulted in 36 PLF video stimuli (average length of 2 s).

#### Procedure

Participants were seated 80 cm from the computer monitor, precisely as described in Experiment 3. They performed an emotion recognition task of roughly 15 min. The trial procedure followed exactly that of Experiment 3 minus the presence of spatially manipulated PLF stimuli. Participants first completed three practice trials, before completing 36 main experimental trials in one block.

### Results

As in Experiment 3, emotion rating responses for each probed emotion scale on each trial were transformed into scores from 0 to 10 (with 0 representing a response of *Not at all* and 10 a response of *Very*). Emotional recognition scores were calculated as the target emotion rating minus the mean of the two nontarget emotion ratings.

RM-ANOVA was used to analyze emotion recognition accuracy with within-subjects factors of emotion (happy, angry, sad) and kinematic manipulation (K1, K2, K3). This revealed a significant main effect of emotion [*F*(2, 80) = 5.46, *p* = .011, η_*p*_^2^ = .12]. Happy expressions were rated most accurately [mean(*SEM*) = 5.04(0.33)], angry expressions were rated least accurately [mean(*SEM*) = 3.82(0.21)] and sad expressions were intermediate [mean(*SEM*) = 4.79(0.27)]. There was also a main effect of the kinematic manipulation [*F*(2, 80) = 4.17, *p* = .019, η_*p*_^2^ = .09], whereby recognition accuracy was highest for K2 [mean(*SEM*) = 4.77(0.18)], and K3 stimuli [mean(*SEM*) = 4.63(0.19)] and lowest for K1 [mean(*SEM*) = 4.26(0.20)].

As in Experiment 3, we found an Emotion × Kinematic interaction [*F*(4, 160) = 18.41, *p* < .001, η_*p*_^2^ = .32]. Emotion-specific recognition scores increased with increasing speed, from K1 through K3, for both angry and happy stimuli; whereas for sad videos, emotion-specific recognition scores increased as speed decreased, from K3 through K1. See Figure 5B for a visual depiction of these results.

Further supporting results from Experiment 3, all videos were most highly rated for the correct target emotion and the same emotion confusions were observed between angry and sad videos. That is, as speed of angry videos decreases they are rated as less angry [*F*(2, 80) = 8.91, *p* < .001, η_*p*_^2^ = .24] and more likely to be confused for sad [*F*(2, 80) = 10.55, *p* < .001, η_*p*_^2^ = 21] and as speed of sad videos increases they are rated as less sad [*F*(2, 80) = 21.06, *p* < .001, η_*p*_^2^ = .35] and are more likely to be confused for angry. No such differences were observed in happy ratings across different kinematic levels of angry videos (*p* = .85), though sad videos, with increasing speed, were marginally more likely to be rated as happy [*F*(2, 80) = 3.89, *p* < .001, η_*p*_^2^ = .09]. This difference is driven by the difference between K1 and K3. Finally, while happy videos were no more likely to be confused for anger when speed was increased (*p* = .54), decreasing the speed of happy videos did elicit increased ratings for sadness [*F*(2, 80) = 16.58, *p* < .001, η_*p*_^2^ = .29].

## General Discussion

### Facial Emotion Production

In a series of four experiments, we demonstrate an important role for movement speed in both the production and perception of facial emotion expressions. Experiment 1 demonstrates the development and validation of a new video-emotion-induction protocol. As well as recording posed expressions, we induced emotion-specific states for happiness, anger and sadness, as indicated by self-reported emotional state ratings. Utilizing a novel analysis pipeline, we analyzed the movement speed of key face actions on a frame-by-frame basis. In line with previous findings regarding the kinematics of emotional expression from whole-body movement (Barliya et al., 2013; Michalak et al., 2009; Sawada et al., 2003), across Experiments 1c and 2, we observed that happiness and anger are characterized by high-speed face actions and sadness by low-speed movements. Importantly, there was, however, systematic variation across face regions. For example, nose movements did not successfully differentiate the three emotions, whereas mouth and eyebrow movements did. In addition, there was also systematic variation across conditions. For example, speed differences relating to mouth movements for posed emotional expressions were driven by happy being faster than sad and angry; whereas for communicative verbal utterances, differences were driven by sad movements being slower than happy and angry. For posed and spontaneous expressions we demonstrated that faster eyebrow movements distinguished angry from happy and sad expressions (where eyebrow movement speed is relatively low). However, for communicative utterances, faster eyebrow movements distinguished happy from angry and sad expressions.

Our data are consistent with current theories whereby the presence of key spatial action units can be used to differentiate facial emotion expressions (Ekman & Friesen, 1977), but highlight an important and neglected role for kinematics. For example, an upturned and downturned mouth differentiates sad and happy expressions. Extending this, we demonstrate that the speed with which the mouth moves into this up/down-turned position is a cue that contributes to emotion recognition independently from the spatial (up/down-turned) position. The current data also converge with studies that have highlighted the importance of particular regions of the face in emotion recognition from dynamic stimuli (Blais et al., 2012; Delis et al., 2016; Jack et al., 2014; Jack & Schyns, 2015). Previous investigations have, for example, shown that the mouth region is the most informative face region with respect to emotion recognition from static displays and the most discriminative region with respect to emotion recognition from dynamic stimuli (Blais et al., 2012). Here we show that the mouth region consistently displays a profile of high-speed movement for happy, slow movement for sad, and intermediate speed for angry. Thus, suggesting that it may be the most informative region with respect to emotion recognition from dynamic displays (see Blais et al., 2012) because its speed reliably differs between the emotions. Parallels may also be drawn with work by Jack and colleagues (Delis et al., 2016; Jack et al., 2014; Jack & Schyns, 2015) that demonstrated that anger expressions could be successfully distinguished from other emotions (specifically disgust) using eyebrow-related cues. In line with this we demonstrated an important role for the eyebrow region in differentiating anger from sadness and happiness. Further work should include emotional expressions such as disgust, surprise, and fear in order to quantify the utility of the eyebrow region in discriminating various different emotions.

With respect to the generalizability of the current findings it is important to consider cultural differences which exist in the production of facial emotion expressions. For example, as previously mentioned, not only is the temporal order of key action units within a sequence diagnostic of specific emotions, but the temporal order has been shown to differ between cultures. For example, Jack and colleagues demonstrated that East Asian individuals use increased early eye region activity to represent emotional intensity, while Western individuals do not (Jack et al., 2012). Thus, it is worth remembering, when generalizing our findings to other groups, that there may also be subtle differences in facial emotion production with regards to facial movement kinematics across distinct cultures.

Nevertheless, data from Experiments 1 and 2 have important implications for expression-centered adaptive gaming and learning platforms that employ facial emotion tracking. The extent to which the kinematics (e.g., speed) of facial expressions, or the rate at which the emotion unfolds across time, carry important information about emotional state is currently unclear. While there have been some attempts to combine spatial and temporal features into facial landmark detection and tracking algorithms (e.g., Pantic & Patras, 2006), many software packages use algorithms (e.g., artificial neural networks; see Yang et al., 2018) to detect the presence and intensity of key facial action units typical for each emotion (Jain et al., 2017; Littlewort et al., 2011). Many such algorithms may benefit from incorporating information about the kinematics of face movements (Kanade et al., 2000; Littlewort et al., 2011; Lyons et al., 1998). A particularly important focus for algorithm development may be the actions that were found to be significant differentiators of emotional state (eyebrow widening, mouth widening, mouth opening, and lip raising).

Importantly, in the current study, mouth movements only differentiated posed and communicative, not spontaneous, emotions. Thus, although mouth movements may be a key cue for detecting the emotional state of another individual with whom we are engaging in a communicative, conversive social interaction, it should be noted that algorithms aiming to detect spontaneous emotions expressed in a context devoid of social interaction, should not rely on posed expression data sets for training purposes. To date, facial landmark detection algorithms, which have considered temporal as well as spatial features of face movement, have tended to focus on posed expressions of facial emotion (e.g., Pantic & Patras, 2006).

More broadly, it is important to consider the context in which automatic emotion recognition software is employed and whether contextual factors are likely to elicit communicative or noncommunicative emotional expressions. Here we demonstrate that kinematic cues may indicate different emotions depending on the context. For example, high-speed eyebrow movements were indicative of anger in posed and spontaneous expression contexts, and happiness in a communicative context. This is particularly relevant for our understanding of how to train expression recognition algorithms; these should be trained in a context-dependent fashion. Further investigation of the combination of presence/absence of spatial action units and of the kinematics of these actions across emotions and cultures may lead to exciting advances in algorithmic emotion-recognition across various contexts.

### Facial Emotion Perception

Experiments 3 and 4 used point light display stimuli of human communicative facial emotion expressions to investigate the impact of manipulating facial expression speed on emotion-specific recognition accuracy. In a discovery (Experiment 3) and replication sample (Experiment 4), we showed that speeding-up facial expressions promotes anger and happiness judgments, and slowing-down expressions encourages sad judgments. Incorporating a cross-factor design to our stimuli generation, whereby three kinematic levels were generated at each of three spatial levels of exaggeration, in Experiment 3, we demonstrate that the influence of kinematics on emotion recognition is dissociable from the influence of spatial cues. That is, there was no significant interaction between spatial and kinematic factors. Thus, the kinematics of facial movements provide added value, and an independent contribution, with respect to emotion recognition. We also tentatively demonstrate that different emotions may benefit more or less from spatial and kinematic exaggeration, with happy and sad expression accuracy relying more on spatial, and sad expression accuracy more on kinematic, information.

These findings complement previous investigations that highlight the importance of the temporal order of emotional facial expression (Blais et al., 2012; Delis et al., 2016; Jack et al., 2014; Jack & Schyns, 2015). Previous work demonstrates that altering the temporal order of facial action unit activation impacts emotion recognition. Going beyond the temporal order of action unit activation, we replicate the findings by Kamachi and colleagues (2013), regarding the role of kinematics in emotion recognition, extending these to naturalistic stimuli. Our study is the first to show that the speed with which specific face actions are activated causally impacts emotion recognition.

Our findings are in line with those from the whole-body emotion recognition literature. Parallel work from Edey and colleagues (2017), concerning body movements, showed that angry and happy (point light) walkers were rated as most emotionally intense when exhibiting high speed movement, while sad walkers were rated as most emotionally intense when presented characteristically slow. Finally, as investigated by Atkinson et al. (2004) from whole-body dynamic emotion expressions, specific emotion confusions were made in the current facial emotion recognition task. While we cannot comment on emotion confusions across the full range of basic emotions, angry expressions were most often confused for sad expressions and vice versa, while happy expressions were neither more likely to be confused for anger or sadness. When considering the same emotions from whole-body point light displays, Atkinson et al. (2004) also found sad point light displays to be more likely confused with anger than happiness, however, whole-body displays of happy and anger were more likely to be confused with one another than with sadness. This may be due to different morphological similarities between these emotions across whole-body and facial movement.

It is important to acknowledge here that, while the current perception task makes an attempt to investigate blended perceptions between emotion categories by inviting participants to rate each stimulus in terms of the intensity of happy, angry, and sad emotions, emotion labels are still used and free responses regarding a participant’s perception of the expressions is not sought. Further investigation and future studies are required to confirm the degree to which kinematic and spatial features map on to precise emotion categories versus simply aiding an individual to pinpoint where, on a hypothetical 3D space of arousal, valence and intensity, a facial expression may lie. Related to this is the important consideration of cultural differences in emotion categorisation, whereby a universally recognisable set of six emotions has been refuted. East Asian cultures appear to exhibit both altered perceptual scanning patterns of facial expressions (Blais et al., 2008) as well as reliable distinction and categorisation of only two of these six emotions, accompanied by category confusion for all other emotions (Jack et al., 2009; Jack et al., 2012). Thus, it would be interesting to observe how differences in emotion categorisation relate to kinematic cues: for example, whether high versus low speed separates the two distinguishable categories of emotion in East Asian cultures.

One caveat is that the current work employed point light representations of spoken sentences with the audio soundtrack removed. It is important to note that, unlike spontaneously induced dynamic facial expressions, expressions during spoken utterances are usually also accompanied by audible speech, which adds another layer of information to aid in emotion perception. For example, commonly considered acoustic parameters which relay information about emotion in speech include frequency, amplitude and tempo (Banse & Scherer, 1996), and while research suggests the visual modality to provide a superior signal to auditory information, combined auditory-visual information has also proved to aid in emotion identification from speech (Most et al., 1993). Thus, removing the audio component from our stimuli may have resulted in lower emotion recognition accuracy scores than those that would have been observed if auditory information had been included. We, nevertheless, felt that it was important to remove audio cues to be confident that performance differences between conditions were attributable to the processing of visual (spatial/kinematic) cues not auditory cues such as pitch.

The current research paves the way for a better understanding of individual differences in facial emotion expression production and perception. For example, individuals with autism spectrum disorder often exhibit differences in facial emotion expression recognition (Brewer et al., 2016; Davies et al., 1994; Macdonald et al., 1989) as well as atypical movement kinematics (Cook et al., 2013; see Cook, 2016 for a review) and other movement-related problems such as dystonia and motor apraxia (Ming et al., 2007). However, to date the extent to which kinematic and emotion recognition differences are linked is unclear. Likewise, Parkinson’s disease is accompanied by well-established differences in whole-body and facial movement (facial bradykinesia; Bologna et al., 2013) and concomitant problems with the production and processing of emotion in faces (Bologna et al., 2016; Ricciardi et al., 2017). It may be that differences, in autism and/or Parkinson’s disease, in production kinematics are linked with increased/decreased use of kinematics cues when processing others’ facial expressions. However, to date the field has lacked a paradigm that can index the influence of kinematic cues on emotion recognition. Thus, ongoing research into motor control, movement kinematics and emotion recognition across such clinical conditions may benefit from paradigms like the one we report here.

In conclusion, speed of facial movement differentiates happy, angry, and sad emotions and contributes to emotion recognition independently of spatial cues. Despite some systematic differences across regions of the face and emotional expression contexts, happy and angry expressions typically show fastest movement, while sad expressions exhibit low speed movement. Moreover, exaggerating these emotion-specific speed profiles leads to improved emotion recognition accuracy. Thus, we see the importance of movement speed in both the production and recognition of facial emotion. In conjunction, speed cues in both production and recognition of facial emotion expressions may serve to guide social interaction partners in understanding and dynamically adapting to one another’s emotional states. We believe these findings to be of importance for both our understanding of typical facial emotion expression as well as in the increasing application of algorithms designed to detect and track facial emotion. Finally, a greater understanding of the spatial and kinematic signatures of emotional expressions, across communicative and noncommunicative contexts, may provide an important framework for the future study of facial emotion in various clinical conditions.

## Supplementary Material

10.1037/emo0000835.supp

## Figures and Tables

**Table 1 tbl1:** Face Action Vectors Used for Speed Analysis Based on Those Reported in Zane et al. (2019)

Face action vector	Calculation
Eyebrow widen	D2 (distance between Points 21 and 22)
Nose lengthen	D5 (distance between Points 27 and 35 + distance between Points 27 and 31) + D6 (distance between Points 31 and 35)
Lip raise	D7 (distance between Points 27 and 50 + distance between Points 27 and 52)
Mouth widen	D8 (distance between Points 48 and 54)
Mouth open	D9 (distance between Points 50 and 57 + distance between Points 52 and 57)
*Note*. Distances between points were calculated according to Equation 1. For consistency with the Zane et al. (2019) article, we calculated face distances D1–D9, though D1, D3, and D4 were not used in the current analysis.	

**Table 2 tbl2:** Experiment 1c Descriptive Statistics Including Mean and Standard Error of the Mean (SEM) for Each Emotion, Face Action and Expression Condition

Variable	*M*	*SEM*
Emotion		
Happy	0.37	0.01
Angry	0.36	0.01
Sad	0.34	0.01
Face action		
Lip raise	0.47	0.02
Mouth widen	0.27	0.01
Eyebrow widen	0.26	0.01
Mouth open	0.39	0.02
Nose lengthen	0.39	0.01
Condition		
Spontaneous	0.36	0.01
Posed	0.36	0.01
*Note*. All values are presented prior to log-transformation in pixels per frame to aid understanding.		

**Table 3 tbl3:** Descriptive Statistics Including Mean and Standard Error of the Mean (SEM) for Experiment 2 for Each Emotion, Face Action and Expression Condition

Variable	*M*	*SEM*
Emotion		
Happy	1.05	0.03
Angry	1.05	0.04
Sad	0.86	0.03
Face action		
Lip raise	1.12	0.04
Mouth widen	0.67	0.02
Eyebrow widen	0.40	0.02
Mouth open	2.07	0.08
Nose lengthen	0.68	0.02
Condition		
Spoken	1.58	0.05
Posed	0.40	0.01
*Note*. All values are presented prior to log-transformation in pixels per frame to aid understanding.		

**Table 4 tbl4:** Mean and Standard Error of the Mean (SEM) for Emotion Recognition at Each Spatial Exaggeration Level (S1, S2, S3) for Each Emotion (Happy, Angry, Sad)

Emotion	Spatial level	*M*	*SEM*
Happy	S1	2.11	0.40
	S2	5.20	0.36
	S3	6.63	0.36
Angry	S1	0.32	0.20
	S2	3.62	0.32
	S3	5.47	0.27
Sad	S1	4.59	0.36
	S2	3.51	0.32
	S3	3.44	0.27

## Footnotes

1 For S1 K3 the spatial manipulation reduced speed by 50% and the kinematic manipulation increased that speed by 50%, thus stimuli are 75% normal speed. Similarly, for S3 K1 the spatial manipulation increased speed by 50% but kinematic manipulation reduced that speed by 50%.
