# Supplementary material for: The Role of Movement Kinematics in Facial Emotion Expression Production and Recognition
Source: Emotion. 2021 Mar 4;21(5):1041–61. doi: 10.1037/emo0000835 (PMC8582590; doi:10.1037/emo0000835)
Supplement: Supplementary file 1 [file EMO-2020-2025_Supplemental_Materials.docx]

**Supplementary materials**

**Supplementary materials A:**

Happiness induction video (Justin Beaver the house beaver) - <https://www.youtube.com/watch?v=DggHeuhpFvg>

Sadness induction video (Eve’s cancer story) - <https://www.youtube.com/watch?v=bWAatejVqzc&t=1s>

Anger induction video (Donald Trump talking about women) - <https://www.youtube.com/watch?v=H6PPB9N8Ax8&t=1s>

Neutral filler video 1 (Homemade pottery lesson) - <https://www.youtube.com/watch?v=A94gHYcwqBo>

Neutral filler video 2 (Paper aeroplane tutorial) - <https://www.youtube.com/watch?v=1PVA2nPU60U>

Neutral filler video 3 (Botanical garden tour) - <https://www.youtube.com/watch?v=mo7BBXCJ3gM&t=2s>

**Supplementary materials B:**

**Instructions for spontaneous production condition:**

We will ask you to sit and watch a series of videos (roughly 3 minutes in duration each) and then to make some ratings after each video. Before the start of each video we will ask you to return your head to the frame positioned on the desk in front of you, so that your chin is in line with the white frame edge. Please hold your head in the frame during the entirety of each video. An instruction on screen will tell you when you can relax between each video.

**Instructions for posed production condition:**

Now we will ask you to imagine you are in a number of emotional states and to pose facial expressions. Please pose each emotion in an exaggerated way, so that someone could watch your video and guess which expression you are posing.

This is the procedure:

1. We will give you as long as you need **to sit and imagine** you are in that emotional state as strongly as you can. When you are ready, position your head inside the white frame and tell the experimenter you are ready to start the recording.
2. You should then listen for the **first beep** with which you should **pose a neutral expression**.
3. You will then hear a second, higher pitched beep to indicate you should now **move your face in your own time from that neutral expression into the facial expression we have asked you to pose**.
4. Please hold your expression until you again hear a lower pitched beep when you should return your face to **neutral** once more.
5. A final long beep will signal that the recording has ended and you can relax.

Here are two examples of exaggerated posed expressions for Disgust and Surprise:

Download example posed disgust video at <https://osf.io/sxm93/>

Download example posed surprise video at <https://osf.io/8jxyd/>

You will have a practise with the emotions surprise and disgust

**Supplementary materials C:**

**Instructions for spoken/communicative production task:**

We will now ask you to imagine you are in a number of different emotional states and to say a sentence whilst moving your face in a way which displays the facial expression for this emotion. When saying the sentence, please exaggerate the expression, so that someone could guess which emotion you are displaying in your face without hearing your sentence.

This is the procedure:

1. We will ask you **to sit and imagine** you are in an emotional state as strongly as you can. Tell the experimenter when you are ready and place your head inside the white frame, facing towards the camcorder.
2. **A beep will then signal when you should start saying the sentence.**
3. The long beep will mean the recording has finished and you can relax.

The sentence we would like you to say:
‘**My name is John and I’m a scientist**’

**Supplementary materials D:**

See example spoken neutral utterance video at <https://osf.io/fmx9p/>

See example spoken disgust utterance video at <https://osf.io/gwxn6/>

See example spoken surprise utterance video at <https://osf.io/ypagv/>

**Supplementary materials E:**

Download example dynamic point light face stimulus angry at <https://osf.io/ta3d7/>

Download example dynamic point light face stimulus happy at <https://osf.io/jwr7y/>

Download example dynamic point light face stimulus sad at <https://osf.io/upzm6/>

**Supplementary materials F:**

Table

*Experiment 3. Mean and standard error of the mean (SEM) for emotion recognition at each kinematic level (K1, K2, K3) at spatial level 2 for each emotion (happy, angry, sad).*

|  |  | Mean | SEM |
| --- | --- | --- | --- |
| Happy | K1 | 4.49 | 0.49 |
|  | K2 | 5.04 | 0.41 |
|  | K3 | 6.08 | 0.38 |
| Angry | K1 | 2.89 | 0.37 |
|  | K2 | 3.28 | 0.37 |
|  | K3 | 4.70 | 0.30 |
| Sad | K1 | 4.45 | 0.47 |
|  | K2 | 4.11 | 0.36 |
|  | K3 | 1.96 | 0.39 |
